# A morphometric analysis of the circumolivary fiber bundle of the human brainstem

**DOI:** 10.3389/fnana.2022.990862

**Published:** 2022-11-16

**Authors:** Victor A. Brendel, Michael J. Schmeisser, Sven Schumann

**Affiliations:** Institute of Anatomy, University Medical Center of the Johannes Gutenberg-University Mainz, Mainz, Germany

**Keywords:** circumolivary fibers, arcuate nucleus, anterior external arcuate fibers, preolivary fibers, laterality, medulla oblongata, anatomical variations

## Abstract

The circumolivary fiber bundle (CFB) is considered to be an anatomical variation, which can be found on the surface of the human medulla oblongata. The macroscopical fiber bundle runs downwards from either the anterior median fissure, the pyramid, or both, around the inferior pole of the olive and turns upwards to reach the restiform body of the inferior cerebellar peduncle. Multiple fiber systems feed the constitution of the CFB (collateral corticospinal fibers, fibers connecting to the reticular formation, anterior external arcuate fibers). With this examination we provide a systematic analysis of the frequency of occurrence (6.14%), size, and laterality of the CFB. Including all three fiber bundle parts (descending part, genu, and ascending part), the left-sided sizes were increased. Likewise, the appearance of an unilateral left-sided CFB could be detected in more than 60% of our cases. Our morphometrical analysis currently covers the largest sample of investigated brainstem sides (*n* = 489) so far. This investigation should widen the perspective on how anatomists, neuroradiologists, and neurosurgeons expect the anterolateral surface of the human medulla oblongata.

## Introduction

Almost 160 years ago Paul Pierre Broca initiated the beginning of intensive research and discussions about a prime example of laterality in the brain: hemispheric dominance (Samara and Tsangaris, 2011; Güntürkün et al., 2020). When he said the famous words: “Nous parlons avec l’hémisphère gauche” or “We speak with the left hemisphere” (Broca, 1865, p.384), he did not know that a few decades later the term “laterality” might develop into a multidimensional construct. In humans as well as in non-human species various lateralities of functional (e.g., motor lateralization including manual and vocal skills, sensory lateralization) and neuroanatomical systems could be found (Fitch and Braccini, 2013; Corballis and Häberling, 2017). Anatomical variations can be defined as the presentation of body structures with morphological features different from those that are described in the majority of individuals. A wide number of those variations of the brain can be found in literature (e.g., Cavum vergae, absence of the interthalamic adhesion). In several cases, anatomical variations influence the laterality of the body, including the brain. Anatomical regions of the left hemisphere of the human brain, which are included in language processing show larger dimensions. For example, an increased size of the temporal planum, which is part of Wernicke’s area, can be detected in different studies (Samara and Tsangaris, 2011; Corballis, 2013); but focusing on brainstem structures, asymmetries can only be found in the human species (Baizer et al., 2022).

Circumolivary fibers or anterior external arcuate fibers are to be understood as fibers on the ventrolateral surface of a human medulla oblongata. The fibers run downwards from either the anterior median fissure, the pyramid, or both, run around the inferior pole of the olive and turn upwards to reach the restiform body of the inferior cerebellar peduncle (Smith, 1904; Hajós, 1914; Swank, 1934; Juba, 1935; Marburg, 1945; De Caro et al., 1998). These fibers can form a macroscopically visible bundle on the brainstem.

Multiple fiber systems feed the constitution of the circumolivary fiber bundle (CFB). Fibers originating from the pyramid, swinging around the inferior part of the olive and fusing with the inferior cerebellar peduncle (ICP) can be described as collateral corticospinal fibers (Swank, 1934; De Caro et al., 1998). The exact termination of the fibers remains uncertain (Smith, 1904; Hajós, 1914; De Caro et al., 1998; Stonebridge et al., 2020).

Fibers located more superficially are described as anterior external arcuate fibers (AEAF; Marburg, 1945; Rasmussen and Peyton, 1946; Stonebridge et al., 2020). The most ventral paired nuclei of the medulla oblongata, the arcuate nuclei (AN) send axonal outgrowths along the same general path of the CFB. The connections of the AN and its function is intensively discussed in literature. Together with other functional centers of the medulla oblongata the AN might be integrated in breathing control and cardiorespiratory mechanisms (Zec et al., 1997; Matturri et al., 2004; Fu and Watson, 2012; Baizer, 2014; Paradiso et al., 2018; Stonebridge et al., 2020). Finally, anterior fibers reaching the reticular formation of the medulla oblongata have been described and added to the circumolivary fiber bundle as well (Swank, 1934; Marburg, 1945; Stonebridge et al., 2020). Fibers need to be noted, which originate from cell groups of the paramedian tracts. In humans the exact termination of those fibers is not known. In macaque brains the nucleus pararaphales sends fibers around the inferior pole of the olive and let them terminate in floccular and parafloccular regions of the cerebellum. These potential connections might play a role in affecting vertical eye-movements and if damaged, might trigger a gaze-evoked-nystagmus (Büttner-Ennever and Horn, 1996).

Additional fibers running laterally from the pyramid and covering the olive are described as preolivary fibers. While the CFB encircles the inferior pole of the olive, the preolivary fiber bundle (PFB) covers the lower or middle part of the surface of the olive. Comparing the vertical diameter of the olive of the brainstem side which shows PFB with the contralateral olive, the contralateral diameter appears enlarged. This fact is explained by the partial covering of the olive surface by the PFB. After running over the olive surface, the PFB joins the pathway of the AEAF (Barnes, 1901; Smith, 1904; Hajós, 1914; Marburg, 1945; Stonebridge et al., 2020).

The goal of this examination was to give a systematic analysis of the frequency of occurrence, the size, and laterality of the CFB. This analysis might help to decipher the function of the CFB and will provide novel morphometrical information about the brain for anatomists, neuroradiologists, and neurosurgeons. This knowledge could be of clinical importance, for example during surgical therapy of clival tumors.

## Materials and Methods

This study was performed on adult human brainstems from the anatomical collection of the Institute of Anatomy, University Medical Center of the Johannes Gutenberg-University Mainz. All brainstems originated from body donors who donated their bodies willingly for research and education. All brains have been fixed with formaldehyde. In total, 489 brainstem sides of unknown sex, age, and medical history were examined.

**Figure 1:**
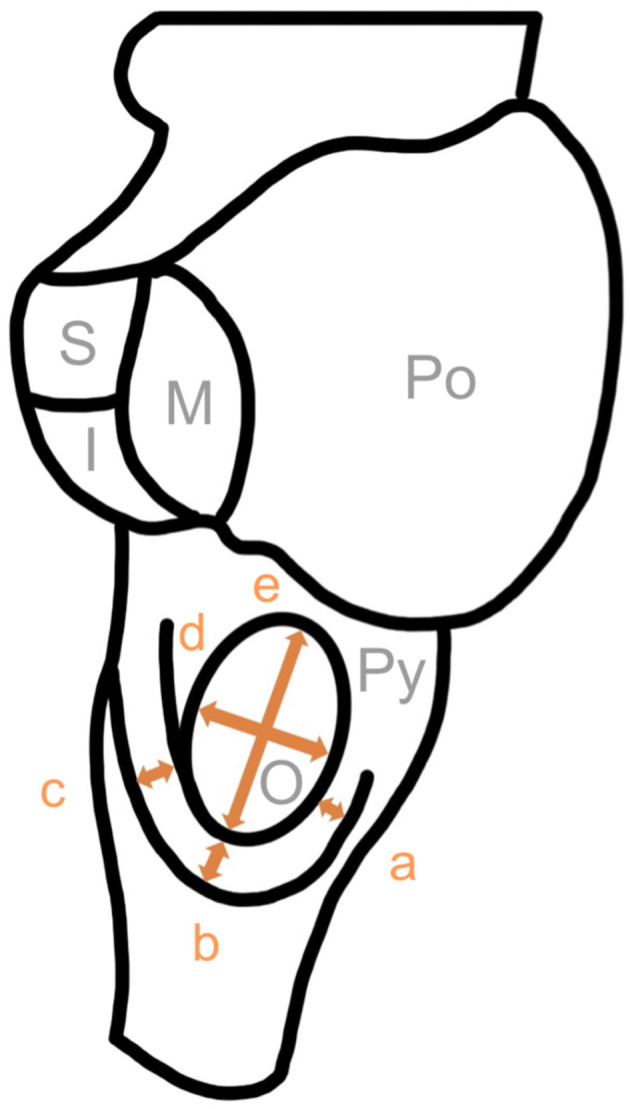
Measure points of the circumolivary fiber bundle and the olive. a, diameter descending part; b, diameter genu; c, diameter ascending part; d, horizontal diameter of the olive; e, vertical diameter of the olive; Py, pyramid; O, olive; Po, pons; S, superior cerebellar peduncle; M, middle cerebellar peduncle; I, inferior cerebellar peduncle.

First of all, we examined all brainstems (complete brainstems and brainstem halves). We defined the circumolivary fiber bundle as a macroscopically visible fiber bundle with a minimum diameter of 0.1 mm. The prevalence and the number of equal-sided fiber bundles were examined. Subsequently predetermined diameters of the CFB and olives were measured using millimeter rulers (Figure 1). The diameters of multiple ipsilateral circumolivary fiber bundles were added together.

Afterwards the additional appearance of preolivary fiber bundles was examined. A fiber bundle was classified as PFB, when it lied on the surface of the olive, and parts of the olive surface were visible below the bundle. If the fiber bundle lied below the inferior border of the olive or covered the inferior border, this bundle was classified as a CFB. Only fiber bundles which showed a minimum diameter of 0.1 mm were included in this study.

To examine the laterality, we investigated only whole brainstems (*n* = 18). We differentiated unilateral and bilateral fiber bundles and counted right-sided and left-sided cases.

This study was conducted according to the guidelines of the Declaration of Helsinki. No further ethical approval was needed for this study.

## Results

**Figure 2:**
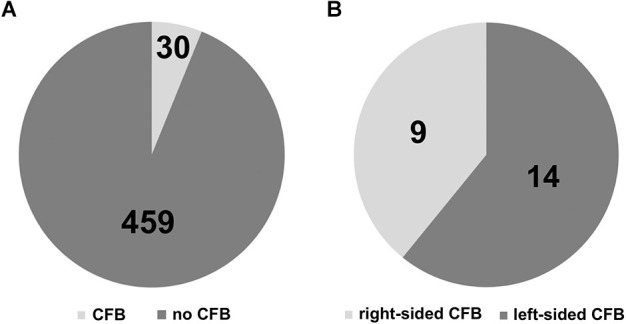
**(A)** Prevalence of circumolivary fiber bundles (CFB vs. no CFB). **(B)** Laterality of the CFB.

**Figure 3:**
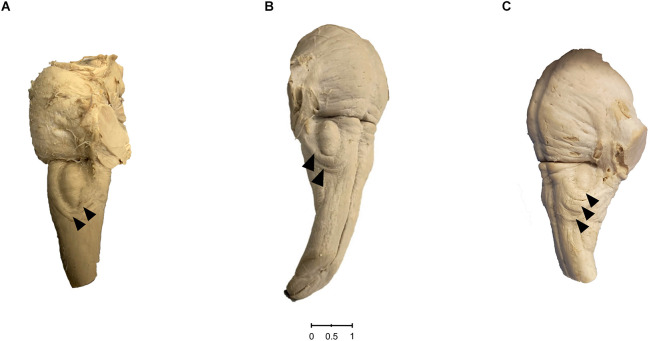
**(A)** Left-sided case of two circumolivary fiber bundles. The photo shows the ventrolateral surface of the human medulla oblongata and pons. The presence of two distinct CFB can be noted on the left side (arrowheads). **(B)** Rare right-sided case of two circumolivary fiber bundles. The photo shows the ventrolateral surface of the human medulla oblongata and pons. The presence of two distinct CFB can be noted on the right side (arrowheads). **(C)** Left-sided case of three circumolivary fiber bundles. The photo shows the ventrolateral surface of the human medulla oblongata and pons. The presence of three distinct CFB with a combined diameter of 7.5 mm (2.5 mm, 3.0 mm, and 2.0 mm) can be noted on the left side (arrowheads).

We found distinct CFB on the surface of 30 out of 489 brainstem sides (Figure 2A). This corresponds to a prevalence of 6.14%. In view of the number of ipsilateral fiber bundles we could observe that the circumolivary fibers of 26 brainstem sides consist of one bundle, three cases show two bundles and on one brainstem side three fiber bundles could be detected. Representative examples of brainstem sides with two CFB are shown in Figure 3A (left-sided CFB) and in Figure 3B (right-sided CFB). The exceptional case of three bundles with a combined diameter of 7.5 mm is shown in Figure 3C.

**Figure 4:**
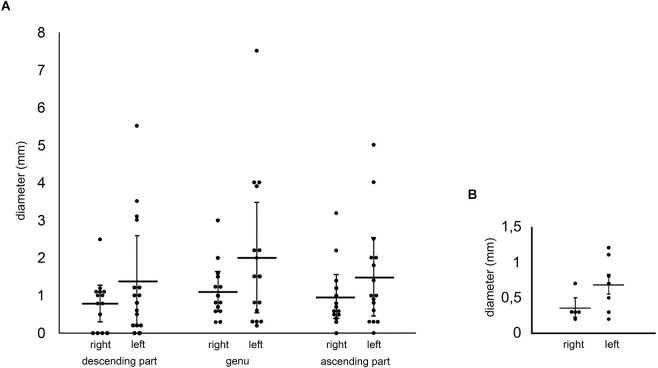
**(A)** Measurements of the circumolivary fiber bundle diameter. Comparisons are made between the right- and left-sided cases as well as between the three parts of the fiber bundle. The diameters of multiple equilateral CFB were added together. **(B)** Measurements of the preolivary fiber bundle diameter. The diagram shows the measurements of the PFB diameter. Comparisons are made between the right- and left-sided cases.

We found differences of the fiber bundle diameter comparing left and right-sided circumolivary fiber bundles (Figure 4A). The means of all three bundle parts (descending part, genu, ascending part) were higher on the left than on the right side. The highest mean was observed in the left genu (2.01 mm), while the lowest mean was detected on the right-sided descending part (0.79 mm). Based on all diameter values we determined a data range from 0.2 to 7.5 mm.

Likewise, we measured the horizontal and vertical diameter of olives of the brainstem sides which showed CFB (43 horizontal and vertical diameters could be measured). On the right side we detected a mean horizontal diameter of 4.85 mm and a vertical diameter of 11.67 mm. A horizontal diameter of 5.16 mm and a vertical diameter of 11.43 mm were detected on the left side. After a direct comparison between left and right-sided diameters, no differences can be pointed out.

However, interesting results were achieved in terms of the presence of additional preolivary fiber bundles. The appearance of five right and seven left-sided cases were observed. Referring to the manual measurements, a difference between right and left brainstem sides was seen again. The mean of the left preolivary fiber bundle diameter was twice the size of the right-sided one (Figure 4B).

We examined the laterality of the circumolivary fiber bundles in three steps. First of all, 18 whole brainstems were divided into two groups: unilateral presence of distinct fiber bundles (*n* = 13) and bilateral appearance of the latter (*n* = 5).

We differentiated four right and nine left-sided cases. Finally, taking a look on the individual sides and including the bilateral cases, nine right and 14 left-sided cases were detected (Figure 2B). This means, in more than 60% the circumolivary fiber bundles were found on the left side.

After comparing the olive diameters, which were determined in connection with the appearance of circumolivary fibers with measurements, which we detected on brainstem sides with no distinct fiber bundles, the following results can be summarized: the mean horizontal right-sided diameter of the olives measured on brainstem surfaces with distinct CFB (5.11 mm) is 0.67 mm bigger than the same diameter of olives on the brainstem surfaces, which did not show any CFB (4.44 mm), the mean vertical left- and right-sided diameter of olives detected on brainstem sided with CFB is reduced by 0.35 mm on the right side (11.89 mm vs. 11.54 mm) and 1.5 mm on the left side (12.63 mm vs. 11.63 mm).

## Discussion

### Prevalence and number of ipsilateral fiber bundles

Hajós (1914), Smith (1904), and Swank (1934) were able to collect several samples of human brainstems showing the circumolivary fiber bundle. Nevertheless, just Swank (1934) mentioned the sample size of investigated human brainstem halves in total (*n* = 170). He did not further classify his population. Our investigations are based on a sample of brainstems originated from one anatomical collection. The brainstems derive from adult German humans (Caucasians, western Europeans).

Swank (1934) detected five unilateral and four bilateral cases resulting in a frequency of 7.65%. The slightly lower prevalence in our investigation (6.14%) can be explained by the approximately three-fold higher number of samples (*n* = 489) and possible differences in the composition of the population. According to the number of ipsilateral circumolivary fiber bundles, we expand the current state of knowledge as well. Most studies up to this date described one single fiber bundle (Smith, 1904; Hajós, 1914; Juba, 1935; De Caro et al., 1998; Stonebridge et al., 2020), Marburg (1945) and Swank (1934) were able to differentiate a doubled left-sided circumolivary bundle. We were able to detect a two-stranded fiber bundle in one-sixth of our cases (*n* = 5) and even observed a left brainstem side, which showed three fiber bundles. However, the appearance of a single-stranded CFB was usually the case (80%).

### Morphometric analysis

Recapitulating all circumolivary bundle diameter values, which were published in earlier works (Smith, 1904; Hajós, 1914; Swank, 1934; De Caro et al., 1998; Stonebridge et al., 2020), a data range of 0.5–5 mm can be summarized (left sided: 0.5–5 mm, right sided: 1.5–3 mm). After summarizing our measurements of the descending part, genu, and the ascending part, we determined an even wider data range on both sides (left: 0.2–7.5 mm, right: 0.3–3.2 mm).

The left-sided sizes of our investigations and of further analyses were determined much wider than the right side (Smith, 1904; Hajós, 1914; Swank, 1934; De Caro et al., 1998; Stonebridge et al., 2020). Additionally, we were able to detect a maximum diameter of 7.5 mm on a left brainstem side, Hajós (1914) just observed a 5 mm thick fiber bundle. On the right side, we determined a maximum diameter of 3.2 mm, which is almost consistent with measurments of De Caro et al. (1998) who detected a 3 mm strong circumolivary fiber bundle. Likewise, in all three fiber bundle parts we were able to measure higher values on the left than on the right brainstem surfaces. This result ties well with previous studies. Consequently, we postulate a more dominant manifestation of the circumolivary fibers on the left brainstem side.

After comparing the olive diameters, which were detected in connection with the presence of circumolivary fibers with those of the brainstem sides, which did not show circumolivary fiber bundles, the left and right-sided horizontal diameter of latter appeared larger. The left sided surfaces of the olives were reduced. Investigations of Smith (1904) underpin these particular results. He observed smaller olives, which were encircled by a circumolivary fiber bundle (Smith, 1904). We anticipate that the olives might be compressed and pulled up the fiber bundle.

To prove a potential loss of olive-volume, imaging techniques or microscopical methods can be added.

According to all manual measurements determined so far, the potential shrinkage of formalin fixated brainstems has not been mentioned in any of the available morphometric studies on the circumolivary fibers. Quester and Schröder (1997) could establish a reduction of 11%–13% on transverse distances, determined on human brainstem surfaces. Longitudinal distances were even reduced by 17%. This means, that the diameter and consequently the volume of the measured fiber bundle parts of the CFB might be increased on native human brainstems. The lowest mean detected on the right-sided descending part (0.79 mm, transverse distance) would be increased by 0.1 mm. Even the highest mean observed on the left genu (2.01 mm, longitudinal distance) would increase to 2.35 mm. If anatomical proportions of brainstem structures should be discussed during neurosurgical imaging processes, this potential shrinkage of 11%–17% must be noticed.

One limitation of this study is the lack of biographic information about the specimens (sex, age, medical history). We did not observe any macroscopically visible pathological changes of the brainstems, but neurological diseases which might influence the morphology of the brainstem could not be excluded completely.

### Preolivary fiber bundle

Five cases of additional preolivary fibers have been mentioned by a few authors (Barnes, 1901; Smith, 1904; Hajós, 1914; Marburg, 1945; Stonebridge et al., 2020). Four left-sided and one bilateral cases were described, just one PFB was measured (Stonebridge et al., 2020). Stonebridge et al. (2020) determined a 1.6 mm thick diameter on each side (bilateral case). We were able to measure 12 cases which showed PFB (seven right, five left sided). Again, we detected a larger dimension of those fibers on the left brainstem side (left-sided mean diameter = 0.69 mm, right-sided mean diameter = 0.36 mm).

### Laterality

Hajós (1914) as well as Smith (1904) established unilateral cases of circumolivary fiber bundles in more than 85% of their cases, Swank (1934) found almost 70% of the circumolivary fibers on the left brainstem side. Furthermore, two bilateral cases (Juba, 1935; Marburg, 1945) and three unilateral right-sided circumolivary fiber bundles (Swank, 1934; De Caro et al., 1998) have to be pointed out. According to our investigations, the circumolivary fibers were also found in approximatly 60% of the cases on the left side. Smith (1904) detected two unilateral right cases out of 60 brainstems; we were able to observe four of such cases out of a reduced sample of 18 brainstems. Nevertheless, we postulate that a unilateral right-sided appearance of circumolivary fiber bundles is less common.

### Arcuate nucleus

For decoding the relevance of the CFB, the integration of the arcuate nuclei and its axonal outgrowths are indispensable (Marburg, 1945; Zec et al., 1997; Stonebridge et al., 2020). On the one hand the paired nucleus is connected with the cerebellum, on the other hand projections terminate in the reticular formation (Marburg, 1945; Zec et al., 1997; Stonebridge et al., 2020). After merging with the restiform body of the ICP, the AEAF migrate medially as medullary striae, pass the raphe and turn ventrocaudally. After crossing to the contralateral side and encircling the ventral surface of the pyramid, the fibers reach the ICP of the other brainstem side (Marburg, 1945; Rasmussen and Peyton, 1946). During microscopical investigations of dominant right-sided medullary striae in conjunction with prominent left-sided AEAF and an intensely stained raphe, Rasmussen and Peyton (1946) substantiated the connection of these structures. The exact fiber connections and functions of the arcuate nuclei still remain to be discovered. Former studies postulate a functional role in breathing regulation and cardiorespiratory circuits (Zec et al., 1997; Matturri et al., 2004; Fu and Watson, 2012; Baizer, 2014; Paradiso et al., 2018; Stonebridge et al., 2020). Fiber tracking studies might underline a potential connection of the arcuate nuclei with the dorsal raphe (Zec et al., 1997). Furthermore, changing the breathing rate of mice after affecting floccular regions, might substantiate a relevant integration mentioned above (Fu and Watson, 2012). There are still reasons to doubt this explanation, because with both investigations a functional integration of a human arcuate nucleus can be speculated, but it cannot be proved (Paradiso et al., 2018).

The morphology of post-mortem investigated arcuate nuclei of infants, who passed away because of SIDS (sudden infant death syndrome) might help to understand the medical relevance of this structure. Matturri et al. (2004) were able to establish a hypoplasia of the arcuate nucleus in approximately 50% of their cases. Paradiso et al. (2018) questioned the theory of AN-hypoplasia but do not negate the potential role in breathing regulation and circulation mechanisms. After investigating the density of infant and adult AN-neurons, the research group determined a loss by more than half of the density values in adult compared to infant cases. The proceeding hypoplasia might be the result of a usual altering process. This could explain the reverse increase of cell groups (e.g., the Pre-Bötzinger complex), which are important for breathing regulation as well. Other authors question this fact and underline the presence of chemosensitive cells in the retrotrapezoid nucleus which is located next to the AN (Lavezzi et al., 2012). Nevertheless, the discussions of the integration of the AN in multiple circuits show the complexity of this nucleus.

Furthermore, there still remains one considerable problem. The human AN is not homologous with the arcuate nucleus of non-human species like mice. Neurobiochemical differences underline this fact. Former studies detected an expression of calbindin-2 (CALB2) in human cases, in arcuate nuclei of mice, however, calbindin-1 (CALB1) was expressed (Fu and Watson, 2012; Stonebridge et al., 2020). Likewise, general asymmetries of brainstem structures are just present in the human species. Brainstems of squirrel monkeys, cats, and mice do not show relevant morphological asymmetries (Berman, 1968; Paxinos et al., 2000; Paxinos and Franklin, 2019; Baizer et al., 2022). The human AN is to be understood as a unique anatomical structure. Investigators of the CFB have to deal with the same issue, since the CFB or a homologous structure has not been detected in animal models.

## Conclusion

In this study, we provide a large-scale morphometric analysis of the circumolivary fiber bundle in a German population. Our morphometrical analysis currently covers the largest sample of investigated brainstem sides.

Based on our examinations the following key points can be outlined:

1. With a frequency of 6.14% (30 out of 489 brainstem sides) the circumolivary fiber bundle (CFB) is a relevant anatomical variation.
2. We were able to determine a larger dimension of the CFB on the left side of the brainstem. Including all three fiber bundle parts (descending part, genu, and ascending part), the right-sided diameter values were unexceptionally reduced.
3. The appearance of an unilateral and bilateral CFB is possible, but in more than 60% we were able to observe the circumolivary fibers on the left side (unilateral).

Taken together, the circumolivary fiber bundle is a common variation of the anterolateral surface of the human medulla oblongata.

## Data Availability Statement

The original contributions presented in the study are included in the article, further inquiries can be directed to the corresponding author.

## Author Contributions

SS conceived and planned the study. VB performed the experiments. VB, MS, and SS discussed the data and wrote the manuscript. All authors contributed to the article and approved the submitted version.

## Conflict of Interest

The authors declare that the research was conducted in the absence of any commercial or financial relationships that could be construed as a potential conflict of interest.

## Publisher’s Note

All claims expressed in this article are solely those of the authors and do not necessarily represent those of their affiliated organizations, or those of the publisher, the editors and the reviewers. Any product that may be evaluated in this article, or claim that may be made by its manufacturer, is not guaranteed or endorsed by the publisher.
